# Physical and Psychological Childbirth Experiences and Early Infant Temperament

**DOI:** 10.3389/fpsyg.2022.792392

**Published:** 2022-03-08

**Authors:** Carmen Power, Claire Williams, Amy Brown

**Affiliations:** ^1^School of Health and Social Care, Faculty of Medicine, Health and Life Science, University of Swansea, Swansea, United Kingdom; ^2^School of Psychology, Faculty of Medicine, Health and Life Science, University of Swansea, Swansea, United Kingdom; ^3^Elysium Neurological Services, Elysium Healthcare, The Avalon Centre, Swindon, United Kingdom

**Keywords:** childbirth experience, infant temperament and behaviour, mother-infant bonding and attachment, postnatal anxiety and depression, post-traumatic stress disorder

## Abstract

**Objective:**

To examine how physical and psychological childbirth experiences affect maternal perceptions and experiences of early infant behavioural style (temperament).

**Background:**

Unnecessary interventions may disturb the normal progression of physiological childbirth and instinctive neonatal behaviours that facilitate mother–infant bonding and breastfeeding. While little is known about how a medicalised birth may influence developing infant temperament, high impact interventions which affect neonatal crying and cortisol levels could have longer term consequences for infant behaviour and functioning.

**Methods:**

A retrospective Internet survey was designed to fully explore maternal experiences of childbirth and her postnatal perceptions of infant behaviour. Data collected from 999 mother–infant dyads were analysed using Pearson’s correlations and multiple analyses of covariance, employing the Bonferroni method of correction to establish initially significant variables. Multiple linear regressions were conducted to determine major perinatal contributors to perceived early infant temperament.

**Results:**

Multiple regression analyses on each of the eight Mother and Baby Scales outcome variables indicated that early infant behavioural style (0–6 months) was largely predicted by subjective maternal states during and post-childbirth, postnatal depression scores, maternal personality traits and infant age. For example, infant age (Beta = 0.440, *p* = 0.000) was the most significant predictor of Alert-Responsive infant behaviour, followed by maternal Postnatal Positive experience (Beta = 0.181, *p* = 0.000). In contrast, depression (EPDS) scores (Beta = 0.370, *p* = 0.000) were the most significant predictor of Unsettled-Irregular infant behaviour, followed by Anxious-Afraid Birth Emotions (Beta = 0.171, *p* = 0.000) and infant age (Beta = −0.196, *p* = 0.000). Mothers also perceived their infants as more Alert-Responsive (Beta = 0.080, *p* = 0.010) and Easier overall (Beta = 0.085, *p* = 0.008) after a Supported birth experience.

**Conclusion:**

Maternal and infant outcomes were influenced by multiple physical and psychological perinatal variables. The mother’s subjective experience appeared to be of equal significance to more objective factors (e.g. birthplace/mode). Social support enhanced the mother’s childbirth experience, benefitting her perceptions of her baby’s early temperament. These findings provide further support for current World Health Organisation intrapartum guidelines (2018) on the importance of making childbirth a ‘*positive experience*’ for women.

## Introduction

Rising levels of childbirth interventions have become a major concern in recent years (Dahlen, 2014; NHS England National Maternity Review, 2016; WHO, 2018). The term ‘childbirth/obstetric intervention’ is used here to refer to any medical interference with the spontaneous physiological progression of ‘normal’ labour and birth, whether due to medical indication, complications or maternal request. While interventions, such as induction and Caesarean section (C-section), were designed to preserve the life and wellbeing of mother and infant, unnecessary interventions may disturb the progression of normal physiological labour and birth, leading to an increased risk of further interventions and complications (Uvnäs-Moberg et al., 2019). This may impede instinctive neonatal behaviours that facilitate mother–infant bonding and breastfeeding post-birth (Widström et al., 2019). Moreover, obstetric interventions increase the risk of the mother developing postpartum depression (PPD) or childbirth-related post-traumatic stress disorder (CB-PTSD; Ayers et al., 2016; Horsch and Garthus-Niegel, 2019).

Therefore, we know that a negative birth experience can have an impact on postnatal maternal mood. Postnatal depression in turn may lead to emotional and behavioural problems in the infant and young child (Murray et al., 2014, 2018). Although infants whose mothers still manage to engage despite their diagnosis can develop well, children are at an increased risk of having behavioural problems aged 3.5 years and cognitive and psychological problems in adolescence if postnatal depression persists (Netsi et al., 2018). Similarly, evidence shows that CB-PTSD may have negative impacts on mother–infant interactions and maternal sensitivity toward her baby (Figueiredo et al., 2008; Parfitt and Ayers, 2009). As well as being very distressing for women suffering from such perinatal psychological disorders, CB-PTSD in the longer term may have negative consequences for the infant’s social–emotional and cognitive development (Garthus-Niegel et al., 2017). Thus, it appears that there could be an indirect pathway between childbirth and infant behaviour *via* postnatal maternal mood.

It is also possible that there are direct links through the inter-connected maternal–infant neurohormonal systems during childbirth (Buckley, 2015; Buckley and Uvnäs-Moberg, 2019). Furthermore, and contrary to previous beliefs (Anand, 2001), we know that the foetus and newborn infant can feel physiological pain and pain-related distress (Anand and Hickey, 1987; Grunau and Craig, 1987; Craig et al., 1993). Certain obstetric interventions, such as assisted birth, have been directly associated with increased levels of neonatal cortisol and crying (Taylor et al., 2000; Gitau et al., 2001). However, comparatively little attention has been given to the possible impacts of childbirth on longer term infant behavioural style, otherwise known as temperament (Thomas and Chess, 1977; Carey and McDevitt, 2016).

Temperament has been defined as ‘*a quality that varies among individuals, is moderately stable over time and situation, is under some genetic influence, and appears early in life—a coherent profile of behavior, affect (emotional state), and physiology (neurochemistry of the brain)*’ (Kagan, 2018, p. 38). It is also ‘*the behavioral style of the individual, the characteristic pattern of experiencing and reacting to the external and internal environment’*. (Carey and McDevitt, 2016, p. 26). These ideas, which together describe temperament as an interaction between genes and the environment, have become widely accepted. Thus, temperament appears to be based on a combination of biological and psychological or experiential substrates.

Assessing infant temperament independently is an intensive activity and therefore research often relies on maternal self-report, although the mother’s mental health post-birth might affect actual or perceived infant temperament. The potential disruptive impact of maternal PPD on normal mother–infant interactions (Murray et al., 2014, 2018; Matthies et al., 2017) may disturb the development of an enduring positive relationship (Feldman, 2017) and subsequent infant behaviour (Feldman et al., 2009). Conceivably, an unsettled infant might exacerbate any maternal mental health issues (Britton, 2011), further affecting mother–infant relationships and longer term infant behaviour.

As well as the finding that newborn infants up to 8 weeks are more likely to be unsettled after an assisted birth (using forceps or ventouse extraction) or emergency C-section (Taylor et al., 2000; Gitau et al., 2001), some authors (Dahlen et al., 2013; Douglas and Hill, 2013) have further suggested that birth complications could affect longer term infant temperament due to the subsequent increase in maternal and foetal cortisol levels (Gitau et al., 1998). This ‘stress response’ may over-stimulate the neonatal hypothalamic–pituitary–adrenal (HPA) axis, with potentially long-term emotional and behavioural consequences for the infant (Dahlen et al., 2013; Douglas and Hill, 2013).

Nevertheless, research exploring how childbirth may (directly or indirectly) affect early infant behaviour and developing temperament is sparse. Our previous qualitative research explored maternity care providers’ perceptions of how this might occur (Power et al., 2019). Our findings highlighted that, while infants may react directly to physical birth events, such as induction, they could also be responding to their mother’s subjective birth experience. Infants who experienced obstetric complications or interventions were perceived as more challenging to care for after birth and often required more comforting. Furthermore, newborns whose mother was distressed or overwhelmed by the birth were also more likely to be unsettled, reflecting her emotional state. This was a possibility originally suggested by Taylor et al. (2000) who argued that a mother’s psychological reaction to the birth could potentially mediate her infant’s crying and stress response to inoculations at 8 weeks after an assisted birth.

This suggestion led to the current study, which aimed to examine whether mothers’ physical and psychological experiences of pregnancy, childbirth and the early postnatal period are associated with perceptions of their infants’ behavioural style, while also considering broader maternal demographic factors, personality and postnatal mood.

## Materials and Methods

### Design

A retrospective online survey examining physical and psychological experiences of childbirth, maternal mental health and infant behaviour.

### Participants

Mothers were eligible to participate if they were over 18 years of age, had an infant aged 0–30 weeks from a singleton pregnancy, resided in the United Kingdom and had no major health problems. Exclusion criteria were: any major health problems in mother or infant; premature birth (<37 weeks); multiple birth (>1 infant) or low birthweight (<5.5 lb) (WHO, 2022).

### Measures

Participants completed an anonymous online survey about their physical and psychological experiences of pregnancy, childbirth and the early postnatal period, alongside a validated measure of perceived infant behavioural style. The survey included items examining the following criteria.

#### Maternal and Infant Demographic Factors

Maternal age, ethnicity, education, postcode, monthly household income and relationship status were reported by mothers, as well as parity (number of children), infant age, gender, gestational age and birthweight (if known).

#### Physical Perinatal Factors

Birthplace (hospital, midwife led unit, home), how labour commenced (e.g. induction or spontaneous) and progressed (e.g. acceleration), timings for each labour stage (hours/minutes), birth interventions (e.g. foetal scalp electrode), birth mode (normal, assisted, planned or emergency C-section), pain ratings, pain relief methods (e.g. ‘gas and air’, pethidine, epidural, water and hypnobirthing) and pre/postnatal complications (e.g., infection, urinary retention) (Stephansson et al., 2016). Items also examined complications, such as foetal distress, meconium in the waters or resuscitation, alongside whether their baby’s head was born gently, whether they had immediate skin-to-skin contact and how they first fed and currently feed their baby (breast, expressed or formula).

#### Psychological Perinatal Factors

Mothers reported how they felt physically and emotionally during pregnancy, childbirth and the postpartum period (e.g. how happy, fearful, energetic or vulnerable they felt). Responses were captured *via* five-point Likert scales (1 = strongly disagree to 5 = strongly agree). Many validated psychological tools, such as the Edinburgh Postnatal Depression Scale (Cox et al., 1987) and the State and Trait Anxiety Inventory (Spielberger et al., 1970), use four-point Likert scales. Thus, the Likert scale is a typical response scale in similar questionnaires that allows for variation between responses to be fully explored.

The subjective measures of pregnancy, birth and postnatal experiences were based on the literature around women’s psychological responses to childbirth and the perinatal period. To the best of our knowledge, at the time of designing this survey, there was no single validated tool that could measure the mother’s actual experience and emotional responses to her experience throughout the perinatal period. Indeed, although there are some validated tools relating to maternal interpretations of the birth experience, such as the Birth Satisfaction Scale (Martins and Fleming, 2011), which examines the mother’s satisfaction with the birth and her care, and a tool by Siassakos et al. (2009) which looks at women’s perceptions of their *operative* birth experience, no one tool covered the period with the breadth required. Furthermore, while maternal satisfaction with the birth, including operative birth experiences and the care she receives, are very important, we were specifically interested in the wider potential impacts of the mother’s *physical* and *psychological* responses to her pregnancy, birth and postnatal experiences on her baby. Consequently, we developed our own questions with a focus on how the mother felt during each stage of her journey, which we could then compare with her perceptions of her baby’s early behavioural style.

#### Infant Behaviour

Infant behaviour was measured using the Mother and Baby Scales (MABS; Wolke and James-Roberts, 1987). This 63-item questionnaire assesses maternal confidence and self-efficacy alongside the mother’s perceptions of specified infant behaviours over the past 7 days. Participants respond *via* six-point Likert scales (0 = not at all to 5 = often/very much) to items, such as ‘*My baby has settled quickly and easily*’ and ‘*During feeds my baby has tended to fuss and cry*’. The scale contains eight sub-scales: Alert-Responsive (A-R); Unsettled-Irregular (U-I); Easiness (E); Alertness during Feeds (ADF); Irritable during Feeds (IDF); Lack of Confidence in Caretaking (LCC) and Breastfeeding (LCBF); and Global Confidence (GC) (see Table 1 for definitions and distribution of MABS scores).

**Table 1:** Mother and baby scales (MABS) and distribution of scores.

MABS area of interest (abbreviations)	Description of measure	Mean	SD	Range (min-max)
**General**	**General categories**			
Alert-responsive (A-R)	Infant alertness, attentiveness and communicativeness with caregivers	39.27	5.13	30.00 (16–46)
Unsettled-irregular (U-I)	Crying/fussing + regularity of eating, sleeping and elimination routines	49.63	12.79	69.00 (19–88)
Lack of confidence in caretaking (LCC)	How capable the mother feels when caring for her baby	32.40	9.12	60.00 (10–70)
**Overall impressions**	**Maternal perceptions of her baby’s behaviour and her own confidence**			
Easiness (easy)	How calm, alert and settled the infant appears overall	24.28	2.30	12.00 (16–28)
Global confidence (GC)	How confident the mother feels about coping; general anxiety level	17.74	1.88	8.00 (13–21)
**Feeding**	**Infant behaviour during feeding**			
Alert during feeds (ADF)	Alertness during feeds	17.09	4.46	23.00 (6–29)
Irritable during feeds (IDF)	Whether the infant feeds reluctantly or with difficulty or irritability	19.18	7.05	39.00 (7–46)
Lack of confidence in breastfeeding (LCBF)	If breastfeeding, whether experienced as problematic (e.g. tension, conflicting advice, technique and birth impacts)	16.49	6.27	38.00 (7–45)

*N* = 999 for all MABS items except lack of confidence in breastfeeding where it is 855.

The MABS have high levels of reliability and validity (Wolke, 1995) and have also demonstrated validity in relation to newer self-report infant temperament questionnaires (Oates et al., 2018). While originally designed for newborn infants, research has employed the MABS with infants aged 0–6 months (Oates et al., 2018), as well as older infants (Field et al., 2002), and in research exploring relationships between infant behaviour, maternal confidence, postpartum depression and low-self-esteem (Denis et al., 2012).

#### Maternal Personality and Postnatal Wellbeing

Heritability factors are known to play an important role in personality development and subsequent behaviour (McAdams and Olson, 2010), and postnatal maternal mood is known to affect infant behaviour (Glover et al., 2018). Therefore, three measures of maternal trait, state and postnatal mood were collected:

- Maternal personality was measured using the Ten Item Personality Inventory (TIPI). The TIPI (Gosling et al., 2003) is a short version of Goldberg’s original hundred-item Big Five Inventory (1992) that self-assesses the personality traits of Extroversion, Conscientiousness, Openness (to new experiences), Agreeableness and Emotional Stability *via* seven-point Likert scales (1 = ‘disagree strongly’; 7 = ‘agree strongly’). Gosling et al. (2003) established the TIPI’s construct validity and test–retest reliability, and it has been widely used in public health research (e.g. Johnston and Brown, 2013).
- Maternal mental health was measured using the 10-item Edinburgh Postnatal Depression Scale (EPDS; Cox et al., 1987) which assesses symptoms of maternal postnatal mood disorder over the previous 7 days. The EPDS presents 10 multiple choice statements (e.g. ‘*I have been able to laugh and see the funny side of things*’), with check box answers on a four-point Likert scale (1 = As much as I ever could/did; 4 = No never/hardly at all). Mothers with scores of 13 or above are considered high risk. The sensitivity, internal consistency, validity and reliability of the EPDS as an effective screening instrument for postnatal depression are well established, and it is widely used in both research and clinical practice in the United Kingdom and elsewhere (e.g., Shrestha et al., 2016).
- Current maternal anxiety state may have affected the mother’s interpretation of childbirth and her baby’s behaviour. Therefore the six-item short form of the ‘State’ scale of Spielberger’s State and Trait Anxiety Inventory (STAI; Spielberger et al., 1970) was included to measure mothers’ current anxiety levels (Marteau and Bekker, 1992). This short version of the STAI State includes six short statements regarding emotional state (e.g. ‘*I feel calm/tense/content/upset*’) on a four-point Likert scale (1 = not at all; 4 = very much). Positive items are reverse scored and therefore higher scores are indicative of higher anxiety levels. This tool is considered a reliable and valid measure of anxiety states (Marteau and Bekker, 1992).

### Procedure

This study was designed and implemented in accordance with the ethical standards of the Declaration of Helsinki, developed by the World Medical Association (2018). A University Department of Psychology Research Ethics Committee granted ethical approval for the study.

Participants were recruited between June 2014 and March 2017 *via* advertisements on United Kingdom mother–infant sites (e.g. bounty.com) and social media (e.g. Facebook and Twitter). The study took place on SurveyMonkey®. On accessing the anonymous online survey, participants were presented with an information page outlining the purpose of the study and eligibility criteria, as well as data protection and confidentiality arrangements. Following electronic consent, participants were asked to complete standard demographic information before beginning the survey. Participation took approximately 15–30 min. Afterwards, participants were thanked and presented with a debrief page outlining where to seek further information and professional support if needed.

#### Data Analysis

Raw data were imported from SurveyMonkey™ into SPSS version 26 (SPSS United Kingdom Ltd). Each of the four questionnaires incorporated in the survey was first scored according to their individual instructions. Multiple statements concerning pregnancy or postnatal complications were summed and included as continuous scores rather than individual items. Continuous, nominal and ordinal data could then be quantified and interpreted *via* the associated tools for analysis: correlations and ANOVAs to establish similarities, differences and interactions of infant behaviour in relation to the birth experience and surrounding factors. Finally, multiple linear regressions were used to establish predictors. Our analysis plan is further detailed below.

To begin, factor analyses were carried out on participant-rated statements concerning their subjective perceptions of the perinatal experience. This technique was applied where multiple statements rated on a five-point Likert scale had the potential for reduction to fewer items: subjective maternal physical and psychological experiences of pregnancy, childbirth and the early postnatal period and overall maternal perceptions of the birth experience (e.g. positive/supported/directed). Mothers had responded to questions and a list of potential answers concerning their personal perceptions of the perinatal period (e.g. ‘*How did you feel during pregnancy/your birth*’). Principal components analyses (PCA) were conducted using Direct Oblimin rotation methods, as recommended by Field (2009) for inter-correlated socio- or psychological data. Factors with an eigenvalue over one were used; computed factors were saved as regression scores, named and used in subsequent data analyses.

To determine which confounding variables required controlling for, Pearson’s bivariate correlations (with two-tailed hypotheses) and Multivariate Analyses of Variance (MANOVAs) were first performed on the sociodemographic and infant characteristics data. Thus, infant characteristics (current infant age, gender, gestational age at birth and birth weight) and sociodemographic variables (maternal age, education, ethnicity, household income, relationship status and number of children) were considered. Significantly associated maternal and infant factors were subsequently controlled for in all further statistical analyses.

Dummy coded variables (yes = 1, no = 0) were created in SPSS where required. Pearson’s partial correlations, controlling for covariates and excluding cases listwise, were conducted where independent variables reflected either ratio or interval data. MANCOVAs were carried out for categorical independent variable data, using the *a priori* Bonferroni correction method of analysis, with all means comparisons chosen in advance (Howell, 2012, 2016). This method of planned comparisons produces equal or slightly more conservative results to planned *post-hoc* tests, while having the advantage of allowing covariates to remain in the equation. An alpha level of <0.05 was used to assess the results of all correlations and MANOVAs.

Monthly household income was categorised using the five income brackets taken from the Office for National Statistics (ONS) division of quintiles from 2014 to 2016, corresponding with the survey design and data collection period (Office for National Statistics, 2017). For multiple linear regression analyses, household incomes were further divided into dichotomous variables: < or >£2,700/month, corresponding to the approximate median gross household income of £2,700/month in the same period (Office for National Statistics, 2019).

Certain factors had the potential to be bi-directional in causality. However, placing perceived infant behaviour and maternal confidence as the speculated outcome variables was integral to the overall study aims: to explore how physical (objective) birth events, psychological variables and the subjective maternal experience of childbirth may influence mother-reported infant behaviour. A predictive form of analysis was chosen to establish the strongest independent variables and to indicate which factors might explain the greatest proportion of the total variance in infant behavioural scores and maternal confidence when all other factors were held constant. Multiple linear regression was used with the forced entry method, excluding cases pairwise to maximise data retention.

Finally, therefore, multiple linear regressions were performed for each of the eight MABS items. In line with Field (2009), multicollinearity was managed in a second regression run for each outcome variable to reduce the inherent inter-correlations often found in psychological data (Field, 2009). Given the large sample size, outliers were only removed if they had an exceptionally large residual (over 30), a leverage value greater than three times the average, or were considered to significantly influence the regression line. Cook’s distance was then employed as a measure of outlier influence and interpreted as satisfactory when <1 in all remaining cases. The Durbin–Watson test was used to establish independence of residuals (Field, 2009). The adjusted *R*^2^ is reported throughout.

## Results

Initially, 1,152 mothers completed the survey although 153 did not meet the inclusion criteria, leaving 999 in the analysis. Mean maternal age on completion of the survey was 32 years (*SD* = 4.2; range 19–44 years); mean infant age was 15.31 weeks (*SD* = 7.48; range 0–30 weeks). Table 2 presents further demographic information.

**Table 2:** Maternal demographic background.

Indicator	Group	*N*	%
Age	19–24	48	4.8
	25–29	217	21.7
	30–34	454	45.4
	35–39	243	24.3
	40–44	32	3.2
Ethnicity	White (British/Irish/Other)	948	94.9
	Mixed/Multiple ethnic group	20	2.0
	Asian/Asian British	13	1.3
	Black African/Black Caribbean	11	1.1
	Other ethnic group	4	0.4
Education (highest level)	No formal qualifications	2	0.2
	GCSE or equivalent	32	3.2
	A level or equivalent	108	10.8
	Degree or equivalent	450	45.0
	Vocational qualification	45	4.5
	Postgraduate or equivalent	361	36.1
Relationship status	Single	19	1.9
	Partner (not living with)	6	0.6
	Cohabiting	261	26.1
	Married	712	71.3
Number of children	1	544	54.5
	2	346	34.6
	3	81	8.1
	4	21	2.1
	5+	4	0.4
Household income^*^	Less than £1,000/month	25	2.5
	£1,000–£1700/month	103	10.3
	£1701–£2,700/month	229	22.9
	£2,701–£4,200/month	335	33.5
	£4,201 or more/month	206	20.6
United Kingdom area of residence	England	735	73.6
	Wales	130	13.0
	Scotland	67	6.7
	Northern Ireland	27	2.7

* Gross household income brackets before tax and after benefits or savings (Office for National Statistics, 2017). Actual percentages (%) are reported for each demographic variable. Where percentages do not total 100, the discrepancy is due to missing data.

### Infant Behaviour

For inclusion in analyses, participants must have completed the Mother and Baby Scales (MABS)—999 mothers who met all the inclusion criteria completed the scale, although only 855 completed the breastfeeding section, corresponding to the breastfeeding data. The MABS data were analysed and coded according to instructions (Brazelton and Nugent, 1995).

Associations between the MABS scores, infant characteristics and maternal demographic background were explored. Infant Age, Infant Gender, Gestational Age, Birth Weight, Maternal Age, Maternal Education and Number of Children were significantly associated with MABS scores and therefore controlled for in all further analyses.

### Physical Perinatal Factors, Perceived Infant Behaviour and Maternal Confidence

#### Pregnancy and Postnatal Complications

The number of complications experienced by mothers during pregnancy and postnatally were computed. Altogether, 37% experienced at least one pregnancy complication (mean 0.51; SD 0.78; range 0–5) and 50.3% at least one postnatal complication (mean 0.91; SD 1.20; range 0–7). Partial Pearson’s correlations identified significant positive associations between the number of pregnancy complications and Unsettled-Irregular infant behaviour as well as Lack of Confidence in Caretaking and Breastfeeding. Similarly, Number of Postnatal Complications had significant positive relationships with Unsettled-Irregular and Irritable during Feeds and negative associations with maternal confidence measures (Table 3). Therefore, where more perinatal complications were experienced, infant behaviour was reported as more Unsettled, Irregular and Irritable and the mother felt less confident.

**Table 3:** Pregnancy and postnatal complications, stages of labour and MABS.

Factor	Pregnancy complications	Postnatal complications	Stages of labour		
			Latent stage (h)*n* = 687	Active stage (h)*n* = 714	2nd stage (min)*n* = 750
Alert-responsive	−0.016, *p* = 0.613	0.032, *p* = 0.322	−0.101, *p* = 0.009^**^	0.022, *p* = 0.565	0.029, *p* = 0.438
Unsettled-irregular	0.103, *p* = 0.001^**^	0.066, *p* = 0.041^*^	0.045, *p* = 0.240	0.113, *p* = 0.003^**^	0.099, *p* = 0.007^**^
Lack of confidence in caretaking	0.140, *p* = 0.000^***^	0.082, *p* = 0.011^*^	0.022, *p* = 0.561	0.127, *p* = 0.001^**^	0.104, *p* = 0.005^**^
Easiness	−0.070, *p* = 0.030^*^	−0.009, *p* = 0.789	−0.017, *p* = 0.662	−0.053, *p* = 0.162	−0.049, *p* = 0.188
Global confidence	−0.074, *p* = 0.021^*^	−0.086, *p* = 0.008^**^	−0.044, *p* = 0.255	−0.032, *p* = 0.392	−0.068, *p* = 0.066
Alert during feeds	0.010, *p* = 0.762	−0.009, *p* = 0.774	0.033, *p* = 0.396	0.018, *p* = 0.630	−0.019, *p* = 0.609
Irritable during feeds	0.105, *p* = 0.001^**^	0.086, *p* = 0.008^**^	0.049, *p* = 0.205	0.022, *p* = 0.562	−0.013, *p* = 0.717
Lack of confidence in breastfeeding	0.088, *p* = 0.011^*^	0.140, *p* = 0.000^***^	−0.007, *p* = 0.872	0.095, *p* = 0.019^*^	0.114, *p* = 0.004^**^

* Pearson’s *r*: *p* < 0.05.

** Pearson’s *r*: *p* < 0.01.

*** Pearson’s *r*: *p* < 0.001.

#### Birthplace

Mothers were asked where they had given birth [hospital, midwife led unit (MLU) or home]. A MANCOVA was conducted to highlight any significant differences between birth settings. Bonferroni tests highlighted that infants were rated as less Alert and Responsive after a hospital birth compared to a MLU or home birth: Hospital *M* = 39.07, *SD* = 5.26; MLU *M* = 39.70, *SD* = 4.70; Home *M* = 39.83, *SD* = 4.75 [*F* (2, 799) = 3.258, *p* = 0.039]. Additionally, infants were rated as more Unsettled and Irregular after a hospital or MLU birth than after a homebirth: Hospital *M* = 50.58, *SD* = 12.57; MLU *M* = 50.60, *SD* = 12.56; Home *M* = 45.06, *SD* = 10.13 [*F* (2, 799) = 6.788, *p* = 0.001]. Finally, mothers reported lower Lack of Confidence in Breastfeeding after homebirths rather than hospital or MLU births: Hospital *M* = 17.06, *SD* = 6.61; MLU *M* = 16.43, *SD* = 5.66; Home *M* = 13.58, *SD* = 4.09 [*F* (2, 799) = 6.753, *p* = 0.001].

#### Start of Labour

A series of MANCOVAs were conducted for each start of labour method in relation to MABS. Notably, infants were significantly less Unsettled-Irregular after Spontaneous Labour (*N* = 489) than by any means of induction: Yes Spontaneous Labour *M* = 49.43, *SD* = 12.67; No Spontaneous Labour *M* = 50.67, *SD* = 11.99 [*F* (1, 812) = 3.79, *p* = 0.05]. Infants were also less Alert-Responsive after a Sweep (*N* = 179) than after No Sweep: Yes Sweep *M* = 38.64, *SD* = 5.50; No Sweep *M* = 39.43, *SD* = 5.00 [*F* (1, 812) = 5.77, *p* = 0.016].

Mothers reported *lower* Lack of Confidence in Breastfeeding after Spontaneous Labour: Yes Spontaneous Labour *M* = 15.74, *SD* = 6.01; No Spontaneous Labour *M* = 17.68, *SD* = 6.49 [*F* (1, 812) = 16.87, *p* = 0.000]. Equally, mothers reported *greater* Lack of Confidence in Breastfeeding after a Membrane Sweep: Yes Membrane Sweep *M* = 17.98, *SD* = 7.00; No Membrane Sweep *M* = 16.13, *SD* = 6.01 [*F* (1, 812) = 5.99, *p* = 0.015]. Similarly, they reported greater Lack of Confidence in Breastfeeding when Induced by Pessary and Drip (*N* = 60): Yes Pessary and Drip *M* = 19.45, *SD* = 8.01; No Pessary and Drip *M* = 16.30, *SD* = 6.07 [*F* (1, 812) = 11.26, *p* = 0.001].

#### Duration of Stages of Labour

Pearson’s partial correlations were conducted to assess associations between the length of each labour stage (latent, active and 2nd stage) and MABS scores. Length of Latent Stage was inversely associated with Alert-Responsive infant behaviour, while lengths of Active Stage and Second Stage were positively associated with Unsettled-Irregular infant behaviour and Lack of Confidence in Caretaking and Breastfeeding. Overall, infants were generally less alert, more unsettled and mothers less confident, after a longer labour (see Table 3).

#### Labour Interventions

Mothers responded to a series of questions about interventions that may have occurred during labour. These included whether they had experienced Artificial Rupture of Membranes (ARM), (continuous) Electronic Foetal Monitoring (EFM), Foetal Scalp Electrode (FSE) or a Foetal Blood Sample (FBS). Table 4 highlights significant differences in MABS outcomes between mothers who reported the presence or absence of these interventions. ARM, acceleration of labour, continuous EFM and FSE were linked to an increase in perceived Unsettled-Irregular infant behaviours, while mothers who experienced ARM also reported their infants as being more Irritable during Feeds. In addition, mothers had greater Lack of Confidence in Caretaking and Breastfeeding after ARM, acceleration of labour, continuous EFM and FSM.

**Table 4:** Labour interventions and MABS.

Factor MABS	Labour intervention—M (SD) and significance														
	ARM*n* = 209			Acceleration*n* = 191			EFM*n* = 363			FSE*n* = 159			FBS*n* = 46		
	Yes	No	*Sig*.	Yes	No	*Sig*.	Yes	No	*Sig*.	Yes	No	*Sig*.	Yes	No	*Sig*.
A-R	38.66 (5.66)	39.47 (4.92)	*F* (1,779) = 3.11, *p* = 0.078	39.08 (5.29)	39.32 (5.10)	*F* (1, 790) = 0.38, *p* = 0.537	39.23 (5.06)	39.30 (5.22)	*F* (1,788) = 0.67, *p* = 0.413	39.30 (5.13)	39.30 (5.16)	*F* (1,740) = 0.34, *p* = 0.562	38.80 (4.89)	39.38 (5.07)	*F* (1,728) = 0.14, *p* = 0.708
U-I	51.42 (12.90)	49.29 (12.16)	*F* (1,779) = 4.59, *p* = 0.032^*^	51.62 (12.67)	49.35 (12.32)	*F* (1, 790) = 4.73, *p* = 0.030^*^	51.66 (12.43)	48.67 (12.34)	*F* (1,788) = 11.93, *p* = 0.001^**^	52.01 (11.79)	49.45 (12.71)	*F* (1,740) = 4.74, *p* = 0.030^*^	53.26 (12.87)	49.67 (12.47)	*F* (1,728) = 2.40, *p* = 0.122
LCC	33.49 (9.36)	31.73 (8.30)	*F* (1,779) = 4.67, *p* = 0.031^*^	34.14 (8.50)	31.56 (8.52)	*F* (1, 790) = 5.06, *p* = 0.025^*^	33.67 (8.77)	31.04 (8.22)	*F* (1,788) = 10.16, *p* = 0.001^**^	33.58 (8.79)	31.83 (8.48)	*F* (1,740) = 1.13, *p* = 0.287	35.46 (8.18)	31.90 (8.51)	*F* (1,728) = 4.23, *p* = 0.040^*^
Easy	24.28 (2.33)	24.17 (2.28)	*F* (1,779) = 0.40, *p* = 0.532	24.16 (2.25)	24.20 (2.31)	*F* (1, 790) = 0.09, *p* = 0.761	24.15 (2.17)	24.23 (2.40)	*F* (1,788) = 0.50, *p* = 0.481	24.22 (2.36)	24.20 (2.30)	*F* (1,740) = 0.03, *p* = 0.872	24.09 (2.15)	24.18 (2.30)	*F* (1,728) = 0.01, *p* = 0.927
GC	17.71 (1.85)	17.74 (1.88)	*F* (1,779) = 0.00, *p* = 0.952	17.54 (1.91)	17.80 (1.84)	*F* (1, 790) = 1.09, *p* = 0.297	17.66 (1.88)	17.81 (1.86)	*F* (1,788) = 0.46, *p* = 0.496	17.75 (1.79)	17.78 (1.88)	*F* (1,740) = 0.20, *p* = 0.655	17.22 (2.04)	17.78 (1.87)	*F* (1,728) = 2.39, *p* = 0.122
ADF	16.64 (4.26)	16.90 (4.51)	*F* (1,779) = 0.25, *p* = 0.618	16.86 (4.23)	16.77 (4.50)	*F* (1, 790) = 0.17, *p* = 0.680	17.02 (4.36)	16.59 (4.48)	*F* (1,788) = 1.48, *p* = 0.225	16.80 (4.17)	16.86 (4.47)	*F* (1,740) = 0.17, *p* = 0.679	17.09 (4.49)	16.84 (4.42)	*F* (1,728) = 0.273, *p* = 0.601
IDF	20.07 (7.25)	18.92 (6.62)	*F* (1,779) = 4.09, *p* = 0.044^*^	19.09 (6.80)	19.25 (6.82)	*F* (1, 790) = 0.48, *p* = 0.490	19.43 (7.04)	19.07 (6.70)	*F* (1,788) = 0.10, *p* = 0.750	19.87 (6.82)	19.10 (6.84)	*F* (1,740) = 1.06, *p* = 0.304	20.19 (6.50)	19.15 (6.83)	*F* (1,728) = 0.19, *p* = 0.661
LCBF	18.07 (7.00)	15.87 (5.85)	*F* (1,779) = 12.95, *p* = 0.000^***^	18.43 (6.62)	15.84 (6.02)	*F* (1, 790) = 12.62, *p* = 0.000^***^	17.86 (7.01)	15.43 (5.34)	*F* (1,788) = 19.11, *p* = 0.000^***^	17.35 (7.06)	16.15 (5.97)	*F* (1,740) = 1.90, *p* = 0.169	19.00 (7.17)	16.30 (6.17)	*F* (1,728) = 4.83, *p* = 0.028^*^

MABS factors are defined in Table 1.

* Multivariate analysis of covariance *F* ratios: *p* < 0.05.

** Multivariate analysis of covariance *F* ratios: *p* < 0.01.

*** Multivariate analysis of covariance *F* ratios: *p* < 0.001.

#### Birth Mode

A series of MANCOVAs assessed differences in perceived infant behaviour and maternal confidence according to Birth Mode. Infants were most likely to be Unsettled-Irregular after Assisted Birth (see Table 5). In addition, mothers felt a greater Lack of Confidence in Caretaking after Assisted Birth or Emergency Caesarean Section and greater Lack of Confidence in Breastfeeding after Assisted Birth.

**Table 5:** Birth mode and MABS.

Factor	Mode of birth—mean (SD)				
	Normal*n* = 646	Assisted*n* = 147	Planned C-section*n* = 66	Emergency C-section*n* = 136	Significance
A-R	39. 21 (5.19)	39.68 (4.43)	38.90 (5.55)	39.18 (5.35)	*F* (3, 802) = 0.69, *p* = 0.559
U-I	49.01 (12.14)	52.52 (13.25)	51.43 (12.07)	51.47 (12.61)	*F* (3, 802) = 3.20, *p* = 0.023^*^
LCC	31.18 (8.34)	34.96 (8.69)	31.75 (8.78)	34.57 (8.29)	*F* (3, 802) = 4.352, *p* = 0.005^**^
Easy	24.21 (2.31)	24.42 (2.37)	23.79 (2.20)	24.03 (2.09)	*F* (3, 802) = 1.28, *p* = 0.279
GC	17.85 (1.79)	17.42 (2.07)	17.51 (1.98)	17.51 (1.87)	*F* (3, 802) = 1.56, *p* = 0.196
ADF	16.56 (4.37)	17.56 (4.34)	17.90 (4.95)	16.67 (4.33)	*F* (3, 802) = 2.74, *p* = 0.042^*^
IDF	19.01 (6.45)	20.06 (7.72)	19.53 (7.56)	19.32 (7.48)	*F* (3, 802) = 0.31, *p* = 0.817
LCBF	15.8 (5.7)	19.2 (8.3)	17.0 (5.9)	17.5 (6.1)	*F* (3, 802) = 7.07, *p* = 0.000^***^

MABS items are defined in Table 1.

* Multivariate analysis of covariance *F* ratios: *p* < 0.05.

** Multivariate analysis of covariance *F* ratios: *p* < 0.01.

*** Multivariate analysis of covariance *F* ratios: *p* < 0.001.

#### Pain Ratings and Pain Relief During Labour

Pearson’s partial correlations found that Pain Ratings during labour were positively associated with Unsettled-Irregular infant behaviour and inversely associated with overall infant Easiness. Pain Ratings were also associated with lower maternal confidence ratings—both globally and in relation to caretaking (Table 6).

**Table 6:** Pain ratings, pain relief and MABS.

Factor	Pain ratings and pain relief						
	Pain level*n* = 955	No meds.*n* = 79	G and A*n* = 696	Pethidine*n* = 141	Spinal*n* = 112	Epidural*n* = 224	General anaesthetic*n* = 12
A-R	−0.049, *p* = 0.131	0.021, *p* = 0.506	−0.056, *p* = 0.081	−0.037, *p* = 0.256	−0.018, *p* = 0.572	−0.029, *p* = 0.375	−0.001, *p* = 0.972
U-I	0.142, *p* = 0.000^***^	−0.025, *p* = 0.430	0.073, *p* = 0.022^*^	0.056, *p* = 0.083	0.029, *p* = 0.375	0.109, *p* = 0.001^**^	0.038, *p* = 0.238
LCC	0.085, *p* = 0.009^**^	−0.035, *p* = 0.271	0.047, *p* = 0.141	0.090, *p* = 0.005^**^	0.043, *p* = 0.185	0.156, *p* = 0.000^***^	0.057, *p* = 0.075
Easy	−0.070, *p* = 0.032^*^	0.044, *p* = 0.167	−0.049, *p* = 0.126	0.008, *p* = 0.808	0.034, *p* = 0.292	−0.075, *p* = 0.020^*^	−0.008, *p* = 0.801
GC	−0.073, *p* = 0.026^*^	0.003, *p* = 0.930	−0.033, *p* = 0.299	−0.072, *p* = 0.024^*^	−0.058, *p* = 0.070	−0.037, *p* = 0.249	0.046, *p* = 0.151
ADF	−0.013, *p* = 0.683	0.031, *p* = 0.328	−0.041, *p* = 0.197	0.014, *p* = 0.660	0.012, *p* = 0.710	−0.003, *p* = 0.923	0.034, *p* = 0.294
IDF	0.055, *p* = 0.092	−0.048, *p* = 0.135	0.021, *p* = 0.505	0.068, *p* = 0.034^*^	0.021, *p* = 0.521	0.039, *p* = 0.223	0.029, *p* = 0.374
LCBF	0.016, *p* = 0.657	−0.012, *p* = 0.546	0.073, *p* = 0.035^*^	−0.020, *p* = 0.559	0.072, *p* = 0.038^*^	0.148, *p* = 0.000^***^	0.034, *p* = 0.326

MABS items are defined in Table 1; No meds., no pain relief and G and A, gas and air.

* Pearson’s *r*: *p* < 0.05.

** Pearson’s *r*: *p* < 0.01.

*** Pearson’s *r*: *p* < 0.001.

In terms of pain relief, nitrous oxide (Entonox) was positively associated with Unsettled-Irregular infant behaviour and Lack of Confidence in Breastfeeding; Pethidine was positively associated with Irritable during Feeds and Lack of Confidence in Caretaking and inversely associated with Global Confidence; Spinal Block was associated with Lack of Confidence in Breastfeeding; while Epidural was associated with less perceived infant Easiness, more Unsettled-Irregular behaviour and lower maternal Confidence in Caretaking and Breastfeeding (Table 6).

#### Natural Methods of Pain Relief During Labour and Birth

Partial correlations highlighted significant associations between certain natural methods of pain control and infant behaviour. Hypnobirthing was inversely associated with Unsettled-Irregular, *r* (234) = −0.068, *p* = 0.033. Reflexology during labour was positively associated with perceived infant Easiness [*r* (9) = 0.071, *p* = 0.026] and Acupuncture during labour was inversely associated with Alert during Feeds [*r* (6) = −0.071, *p* = 0.026]. Thus, infants were reported as ‘easier’ after Reflexology and as more ‘relaxed during feeds’ after Acupuncture. However, both Acupuncture (*n* = 8, 0.8%) and Reflexology (*n* = 11, 1.1%) had small sample sizes, undermining the reliability of these findings. Therefore, Reflexology and Acupuncture were excluded from further analyses.

#### Tearing and Episiotomy

A MANCOVA employing the Bonferroni correction explored differences between mothers who had or had not experienced a ‘Tear or Episiotomy’. Notably, Episiotomy was too small a group to include as a stand-alone item (*n* = 4, 0.4%). Although there were no significant differences in perceived infant behaviour, differences in maternal Global Confidence were seen between ‘No Tear’ (*n* = 187) and ‘Tear or Episiotomy’ (*n* = 812): No Tear *M* = 18.20, *SD* = 1.76; Tear or Episiotomy *M* = 17.63, *SD* = 1.86 [*F* (1, 815) = 10.351, *p* = 0.001].

#### Foetal and Neonatal Distress

MANCOVAs were conducted between MABS and foetal or neonatal distress signals. Significant increases were seen for Unsettled-Irregular and Irritable during Feeds after Foetal Distress (Table 7). Maternal confidence scores were also lower after Foetal Distress. No significant differences were seen for any MABS infant behaviour items after Meconium in Waters, although Confidence in Caretaking and Breastfeeding scores were significantly lower. Global Confidence was less after Resuscitation.

**Table 7:** Foetal distress signals, gentle birth of head and MABS.

Factor M (SD)	Foetal distress Yes *n* = 194 M(SD)			Meconium in waters Yes *n* = 173 M(SD)			Resuscitation Yes *n* = 57 M(SD)			Gentle birth of head Yes *n* = 369 other *n* = 630 M(SD)		
	Yes	No	Sig.	Yes	No	Sig.	Yes	No	Sig.	Yes	Other	Sig.
A-R	39.56 (4.84)	39.19 (5.18)	*F* (1, 812) = 0.835, *p* = 0.361	39.39 (4.71)	39.23 (5.20)	*F* (1, 812) = 0.041, *p* = 0.839	39.32 (5.13)	39.26 (5.12)	*F* (1, 812) = 0.027, *p* = 0.870	39.71 (4.91)	38.98 (5.23)	*F* (1, 811) = 4.747, *p* = 0.030^*^
U-I	54.41 (13.22)	48.97 (12.02)	*F* (1, 812) = 21.168, *p* = 0.000^***^	51.21 (13.39)	49.68 (12.20)	*F* (1, 812) = 2.612, *p* = 0.106	52.94 (12.35)	49.75 (12.39)	*F* (1, 812) = 3.811, *p* = 0.051	47.51 (11.77)	51.44 (12.56)	*F* (1, 811) = 16.636, *p* = 0.000^***^
LCC	34.83 (8.90)	31.58 (8.35)	*F* (1, 812) = 12.018, *p* = 0.001^**^	34.23 (8.35)	31.74 (8.51)	*F* (1, 812) = 4.959, *p* = 0.026^*^	32.77 (6.88)	32.12 (8.62)	*F* (1, 812) = 0.647, *p* = 0.421	30.80 (8.03)	32.99 (8.73)	*F* (1, 811) = 5.551, *p* = 0.019^*^
Easy	24.08 (2.32)	24.22 (2.28)	*F* (1, 812) = 0.348, *p* = 0.555	23.96 (2.19)	24.25 (2.31)	*F* (1, 812) = 2.903, *p* = 0.089	23.55 (2.51)	24.24 (2.27)	*F* (1, 812) = 4.245, *p* = 0.040	24.47 (2.29)	24.03 (2.27)	*F* (1, 811) = 6.110, *p* = 0.014^*^
GC	17.19 (1.97)	17.86 (1.81)	*F* (1, 812) = 13.928, *p* = 0.000^***^	17.59 (1.89)	17.77 (1.85)	*F* (1, 812) = 0.849, *p* = 0.357	17.19 (2.18)	17.77 (1.83)	*F* (1, 812) = 4.903, *p* = 0.027^*^	18.08 (1.71)	17.53 (1.91)	*F* (1, 811) = 11.706, *p* = 0.001^**^
ADF	16.53 (4.22)	16.85 (4.45)	*F* (1, 812) = 0.667, *p* = 0.414	16.25 (4.32)	16.90 (4.42)	*F* (1, 812) = 2.888, *p* = 0.090	16.00 (4.03)	16.84 (4.43)	*F* (1, 812) = 1.362, *p* = 0.243	16.75 (4.44)	16.82 (4.40)	*F* (1, 811) = 0.068, *p* = 0.794
IDF	20.74 (8.02)	18.91 (6.51)	*F* (1, 812) = 6.911, *p* = 0.009^**^	19.72 (7.15)	19.13 (6.77)	*F* (1, 812) = 0.898, *p* = 0.344	19.85 (6.29)	19.19 (6.86)	*F* (1, 812) = 0.909, *p* = 0.341	18.52 (6.01)	19.67 (7.27)	*F* (1, 811) = 3.033, *p* = 0.082
LCBF	19.38 (8.14)	15.92 (5.63)	*F* (1, 812) = 14.704, *p* = 0.000^***^	18.65 (7.39)	16.11 (5.95)	*F* (1, 812) = 11.937, *p* = 0.001^**^	17.00 (6.54)	16.50 (6.27)	*F* (1, 812) = 0.451, *p* = 0.502	15.18 (5.22)	17.37 (6.72)	*F* (1, 811) = 13.307, *p* = 0.000^***^

MABS items are defined in Table 1.

* Multivariate analysis of covariance *F* ratios: *p* < 0.05.

** Multivariate analysis of covariance *F* ratios: *p* < 0.01.

*** Multivariate analysis of covariance *F* ratios: *p* < 0.001.

#### Gentle Birth of Head

Mothers were asked to recall how gently their baby’s head had been born on a five-point Likert scale (1 = strongly disagree; 5 = strongly agree). This factor was then transformed into a dichotomous variable: ‘Gentle Birth of Head’ or ‘Other’ (a non-gentle birth of the head). Infants were perceived as more Alert-Responsive, less Unsettled-Irregular and Easier overall after a Gentle Birth. In addition, Gentle Birth of Head led to an increase in Global Confidence scores alongside a decrease in Lack of Confidence in Breastfeeding scores (Table 7).

#### Skin-to-Skin Care

MANCOVAs were conducted to differentiate between infants who did or did not have immediate skin-to-skin contact with their mother post-birth. Infants who experienced immediate ‘Skin-to-Skin’ contact were reported as less Unsettled-Irregular: Yes Skin to Skin *M* = 49.63, *SD* = 12.39; No Skin to Skin *M* = 54.30, *SD* = 11.88 [*F* (1, 812) = 11.826, *p* = 0.001]. Infants were also reported as Easier overall: Yes Skin to Skin *M* = 24.25, *SD* = 2.29; No Skin to Skin *M* = 23.55, *SD* = 2.21 [*F* (1, 812) = 6.491, *p* = 0.011].

Overall, mothers reported less Lack of Confidence in Caretaking if they had experienced immediate skin-to-skin contact with their baby post-birth: Yes Skin to Skin *M* = 31.96, *SD* = 8.55; No Skin to Skin *M* = 34.96, *SD* = 7.72 [*F* (1, 812) = 4.773, *p* = 0.029]. They also reported less Lack of Confidence in Breastfeeding after immediate skin-to-skin contact with their baby: Yes Skin to Skin *M* = 16.37, *SD* = 6.16; No Skin to Skin *M* = 18.90, *SD* = 7.43 [*F* (1, 812) = 8.493, *p* = 0.004].

#### Feeding Method: First Feed

The sample consisted of 882 (88.3%) mothers who initiated breastfeeding and 117 (11.7%) who began feeding by any other method, such as syringe fed, formula or expressed bottle fed. Therefore, to facilitate further analyses, First Feed was dichotomised into two groups: Breastfed (‘breastfed’) and Other (‘expressed’, ‘formula’ or ‘other’). In a MANCOVA for First Feed and MABS, perceptions of Unsettled-Irregular infant behaviours increased if the First Feed was ‘Other’: First Feed Breastfed *M* = 49.56, *SD* = 12.27; Other *M* = 54.06, *SD* = 13.26 [*F* (1, 811) = 5.436, *p* = 0.020].

#### Current Feeding Method

Current Feed responses were likewise dichotomised: ‘Currently Breastfed’ and ‘Other’. The sample consisted of 850 participants (85.1%) who were currently breastfeeding, while 146 (14.6%) were feeding by another method (e.g. formula). A MANCOVA was conducted for Current Feeding Method and MABS. Infants were less Alert during Feeds if currently breastfeeding: Currently Breastfed *M* = 16.73, *SD* = 4.36; Other *M* = 20.05, *SD* = 5.37 [*F* (1, 812) = 5.339, *p* = 0.021]. Mothers also understandably had *lower* Lack of Confidence in Breastfeeding if they were currently breastfeeding their baby: Currently Breastfed *M* = 16.45, *SD* = 6.20; Other *M* = 20.33, *SD* = 8.64 [*F* (1, 812) = 3.901, *p* = 0.049].

### Subjective and Psychological Factors, Infant Behaviour and Maternal Confidence

As outlined in the Data Analysis section, PCA with Direct Oblimin rotation methods were used to analyse multiple subjective statements regarding mothers’ personal experiences of pregnancy, childbirth and the postnatal period, as well as her overall perceptions of the birth experience.

As there was no one validated scale that covered individual maternal responses to the whole perinatal period, subjective statements around the mother’s sense of her own physical and psychological wellbeing during the three major stages (pregnancy, childbirth and the postnatal period) were derived from the literature. Questions relating to women’s subjective birth and perinatal experiences were analysed using principal components analysis (PCA) and explained 62%–68% of the variance for each period, as well as for maternal overall perceptions of her birth experience. This compares well to Foley et al. (2014) validation of the Birth Memories and Recall Questionnaire (The Birth MARQ), which examines the relationship between childbirth memories and postpartum mood disorders, explaining 64% of the variance. It also compares favourably to the Childbirth Questionnaire (Dencker et al., 2010) which accounted for 54% of the total variance, with a focus on maternal satisfaction with the birth rather than on her emotional responses to birth and perinatal experiences. Our results showed good internal consistency and reliability between factors stemming from the PCA (Field, 2009) and were therefore considered fit for use in subsequent analyses in relation to infant behaviour.

Subjective pregnancy states included ‘felt happy and excited/anxious and fearful about the birth’ (labelled Positive Pregnancy Emotions) and ‘had plenty of energy/felt tired and drained’ (labelled Positive Physical Pregnancy). In both cases, a higher score indicated a more positive subjective experience. Subjective birth states included: Positive (‘strong, happy, energised and focused’); Neglected (‘abandoned’ or ‘ignored’); Aware-Alert (‘aware’ and ‘alert’); and Anxious-Afraid (‘anxious, afraid, vulnerable and overwhelmed’) Birth Emotions.

These subjective pregnancy and childbirth factors in relation to MABS are presented in Table 8. Pearson’s partial correlations identified numerous significant associations between subjective experiences of pregnancy, childbirth and the postnatal period and reported infant behaviour. Positive maternal experiences were associated with easier infant behaviour, while negative experiences were associated with more challenging infant behaviour (Table 8).

**Table 8:** Subjective pregnancy states, birth emotions, postnatal states, overall birth experience and MABS.

Factor	Pregnancy states *n* = 981		Birthing emotions *n* = 944				Postnatal states *n* = 945			Overall birth experience *n* = 908		
	Positive emotions	Positive physical	Positive	Neglected	Aware-alert	Anxious-afraid	Postnatal distress	Postnatal positive	Postnatal physical wellbeing	Positive	Supported	Directed
A-R	0.085, *p* = 0.009^**^	0.067, *p* = 0.039^*^	0.189, *p* = 0.000^***^	−0.126, *p* = 0.000^***^	0.064, *p* = 0.052	−0.078, *p* = 0.018	−0.121, *p* = 0.000^***^	0.243, *p* = 0.000^***^	0.058, *p* = 0.079	0.123, *p* = 0.000^***^	0.165, *p* = 0.000^***^	−0.075, *p* = 0.026^*^
U-I	−0.241, *p* = 0.000^***^	−0.202, *p* = 0.000^***^	−0.197, *p* = 0.000^***^	0.148, *p* = 0.000^***^	−0.094, *p* = 0.004^**^	0.297, *p* = 0.000^***^	0.259, *p* = 0.000^***^	−0.208, *p* = 0.000^***^	−0.256, *p* = 0.000^***^	−0.207, *p* = 0.000^***^	−0.112, *p* = 0.001^**^	0.138, *p* = 0.000^***^
LCC	−0.128, *p* = 0.000^***^	−0.111, *p* = 0.001^**^	−0.100, *p* = 0.002^**^	0.136, *p* = 0.000^***^	−0.120, *p* = 0.000^***^	0.196, *p* = 0.000^***^	0.221, *p* = 0.000^***^	−0.110, *p* = 0.001^**^	−0.149, *p* = 0.000^***^	−0.178, *p* = 0.000^***^	−0.104, *p* = 0.002^**^	0.126, *p* = 0.000^***^
Easy	0.156, *p* = 0.000^***^	0.091, *p* = 0.005^**^	0.148, *p* = 0.000^***^	−0.088, *p* = 0.008^**^	0.028, *p* = 0.392	−0.160, *p* = 0.000^***^	−0.164, *p* = 0.000^***^	0.193, *p* = 0.000^***^	0.119, *p* = 0.000^***^	0.143, *p* = 0.000^***^	0.081, p = 0.015^*^	−0.044, *p* = 0.192
GC	0.229, *p* = 0.000^***^	0.171, *p* = 0.000^***^	0.187, *p* = 0.000^***^	−0.079, *p* = 0.016^*^	0.093, *p* = 0.005^**^	−0.205, *p* = 0.000^***^	−0.188, *p* = 0.000^***^	0.213, *p* = 0.000^***^	0.211, *p* = 0.000^***^	0.176, *p* = 0.000^***^	0.089, *p* = 0.008^**^	−0.049, *p* = 0.143
ADF	0.030, *p* = 0.354	0.071, *p* = 0.027^*^	0.068, *p* = 0.037^*^	−0.049, *p* = 0.137	0.019, *p* = 0.558	−0.040, *p* = 0.224	−0.041, *p* = 0.210	0.074, *p* = 0.024^*^	0.058, *p* = 0.076	0.041, *p* = 0.217	0.051, *p* = 0.132	0.015, *p* = 0.658
IDF	−0.172, *p* = 0.000^***^	−0.180, *p* = 0.000^***^	−0.110, *p* = 0.001^**^	0.056, *p* = 0.091	−0.095, *p* = 0.004^**^	0.134, *p* = 0.000^***^	0.136, *p* = 0.000^***^	−0.137, *p* = 0.000^***^	−0.172, *p* = 0.000^***^	−0.092, *p* = 0.006^**^	−0.047, *p* = 0.160	0.034, *p* = 0.318
LCBF	−0.136, *p* = 0.000^***^	−0.048, *p* = 0.163	−0.056, *p* = 0.110	0.165, *p* = 0.000^***^	−0.100, *p* = 0.004^**^	0.200, *p* = 0.000^***^	0.178, *p* = 0.000^***^	−0.070, *p* = 0.048^*^	−0.206, *p* = 0.000^***^	−0.179, *p* = 0.000^***^	−0.072, *p* = 0.045^*^	0.155, *p* = 0.000^***^

MABS items are defined in Table 1.

* Pearson’s *r*: *p* < 0.05.

** Pearson’s *r*: *p* < 0.01.

*** Pearson’s *r*: *p* < 0.001.

Next, Pearson’s partial correlations were conducted between subjective maternal postnatal states, the mother’s overall perceptions of her birth experience and her baby’s temperament. Feeling physically and mentally positive post-birth and having an overall positive, supported experience were associated with maternal perceptions of easier and more settled infant behaviour. Equally, feeing distressed postnatally and having a more directed birth experience overall were associated with more difficult, unsettled infant behaviour (see Table 8). Notably, the factor Maternal Postnatal Distress comprised nine items describing negative emotions, such as anger, guilt, confusion and distress. Conversely, Postnatal Positive comprised eight items describing positive postnatal emotions, such as euphoria, exhilaration, relief and pride.

#### Personality, Postnatal Mood and Current State

Maternal personality (TIPI), postnatal mood (EPDS) and current state (STAI State) were significantly associated with MABS items. For example, a more positive mood, feeling calm and traits, such as Openness to new experiences and Emotional Stability, were associated with easier infant behavioural style (see Table 9).

**Table 9:** Maternal personality (TIPI), postnatal mood (EPDS), current state (STAI State) and MABS.

Measure	Factor	Big 5 personality traits (TIPI) *N* = 953					EPDS total *N* = 922	State anxiety *N* = 947
		Extroversion	Agreeable	Conscientious	Emotional stability	Openness		
MABS	Alert-responsive	0.075, *p* = 0.023^*^	0.095, *p* = 0.004^**^	0.043, *p* = 0.190	0.065, *p* = 0.047^*^	0.129, *p* = 0.000^***^	−0.123, *p* = 0.000^***^	−0.158, *p* = 0.000^***^
	Unsettled-irregular	−0.122, *p* = 0.000^***^	−0.134, *p* = 0.000^***^	−0.162, *p* = 0.000^***^	−0.291, *p* = 0.000^***^	−0.150, *p* = 0.000^***^	0.412, *p* = 0.000^***^	0.396, *p* = 0.000^***^
	Lack of confidence in caretaking	−0.084, *p* = 0.010^*^	−0.050, *p* = 0.128	−0.102, *p* = 0.002^**^	−0.240, *p* = 0.000^***^	−0.008, *p* = 0.814	0.380, *p* = 0.000^***^	0.315, *p* = 0.000^***^
	Easiness	0.082, *p* = 0.012^*^	0.105, *p* = 0.001^**^	0.068, *p* = 0.037^*^	0.165, *p* = 0.000^***^	0.170, *p* = 0.000^***^	−0.172, *p* = 0.000^***^	−0.221, *p* = 0.000^***^
	Global confidence	0.133, *p* = 0.000^***^	0.116, *p* = 0.000^***^	0.149, *p* = 0.000^***^	0.303, *p* = 0.000^***^	0.141, *p* = 0.000^***^	−0.350, *p* = 0.000^***^	−0.335, *p* = 0.000^***^
	Alert during feeds	0.084, *p* = 0.010^*^	0.004, *p* = 0.895	0.138, *p* = 0.000^***^	0.070, *p* = 0.033^*^	0.079, *p* = 0.016^*^	−0.088, *p* = 0.008^**^	−0.072, *p* = 0.028^*^
	Irritable during feeds	−0.091, *p* = 0.005^**^	−0.104, *p* = 0.001^**^	−0.082, *p* = 0.012^*^	−0.209, *p* = 0.000^***^	−0.117, *p* = 0.000^***^	0.310, *p* = 0.000^***^	0.286, *p* = 0.000^***^
	Lack of confidence in breastfeeding	−0.093, *p* = 0.008^**^	−0.052, *p* = 0.142	−0.027, *p* = 0.448	−0.145, *p* = 0.000^***^	−0.013, *p* = 0.716	0.227, *p* = 0.000^***^	0.181, *p* = 0.000^***^

* Pearson’s *r*: *p* < 0.05.

** Pearson’s *r*: *p* < 0.01.

*** Pearson’s *r*: *p* < 0.001.

### Perinatal Predictors of Infant Behaviour and Maternal Confidence

As described above, multiple physical and psychological factors were associated with MABS. The next section examines the factors that best predicted infant behaviour and maternal confidence when all other significant variables were held constant. STAI State was highly correlated with EPDS (>0.7). Given that STAI State probably reflected the mother’s current (postnatal) mood, it was decided that EPDS would remain. Consequently, STAI State was removed in the following analyses to reduce multicollinearity.

#### Regression Analyses for MABS

As multiple physical and psychological factors were associated with MABS, the next section examines the factors that best predicted perceived infant behaviour and maternal confidence. Multiple linear regression analyses were conducted for each MABS item to predict maternal reports of infant behaviour and her own self-reported confidence. Significant perinatal factors for infant behaviour and maternal confidence—as highlighted by the Pearson’s correlations and MANCOVAs described above—were entered into the final regression models.

MABS items were split into infant behaviours (Table 10) and maternal confidence (Table 11). Infant behaviour was predicted by a combination of (current) infant age, maternal demographic background, subjective physical and psychological experiences of childbirth and the postnatal period (e.g. Anxious-Afraid Birth Emotions or Postnatal Physical Wellbeing) and psychological factors (e.g. EPDS scores), while more objective physical factors, such as Number of Postnatal Complications, did not remain significant in the final regression model. For example, Alert-Responsive infant behaviour was predicted by higher infant age, maternal openness, lack of maternal higher education, having a supported experience and feeling positive post-birth (Table 10). Therefore, infants generally grew more settled, alert and responsive with increased age, regardless of their birth experience, although infant ‘easiness’ or how unsettled they were perceived as was still affected by maternal mood (EPDS scores) and personality, as can be seen in the regression table for infant behaviour (Table 10).

**Table 10:** Predictors of infant behaviour (0–6 months).

MABS	Variables	Unstandardised coefficients		Standardised coefficients	*t*	*p*
		*B*	*SEB*	*β*		
Alert-responsive	Constant	33.941	0.825		41.165	0.000
	Infant age	0.302	0.020	0.440	14.884	0.000
	Higher education	−1.198	0.432	−0.082	−2.772	0.006
	Postnatal positive	0.929	0.161	0.181	5.784	0.000
	Experience supported	0.409	0.159	0.080	2.565	0.010
	Maternal openness	0.348	0.132	0.079	2.642	0.008
Unsettled-irregular	Constant	48.555	1.040		46.695	0.000
	Infant age	−0.331	0.051	−0.196	−6.653	0.000
	Anxious-afraid BE	2.180	0.419	0.171	5.204	0.000
	Number of PN complications	−0.323	0.330	−0.031	−0.981	0.327
	EPDS total	0.902	0.079	0.370	11.400	0.000
Easiness	Constant	22.958	0.434		52.900	0.000
	Infant age	0.054	0.010	0.176	5.476	0.000
	Higher education	−0.547	0.213	−0.083	−2.576	0.010
	Postnatal positive	0.306	0.078	0.133	3.923	0.000
	Birth partner and birth companion	0.551	0.208	0.085	2.645	0.008
	Maternal openness	0.244	0.065	0.124	3.754	0.000
	EPDS total	−0.046	0.015	−0.104	−3.054	0.002
Alert during feeds	Constant	14.785	0.836		17.692	0.000
	Infant age	0.139	0.018	0.234	7.566	0.000
	Higher education	−1.000	0.401	−0.078	−2.494	0.013
	Breastfed currently	−1.904	0.396	−0.151	−4.810	0.000
	Conscientiousness	0.498	0.119	0.129	4.183	0.000
Irritable during feeds	Constant	24.086	1.603		15.026	0.000
	Infant age	−0.179	0.030	−0.190	−6.035	0.000
	Birth weight	−1.363	0.419	−0.102	−3.254	0.001
	PN physical wellbeing	−0.697	0.233	−0.099	−2.991	0.003
	EPDS total	0.380	0.045	0.279	8.443	0.000

B, unstandardised coefficient; SEB, standard error of unstandardised coefficient; *β*, standardised beta; BE, birth emotions; PN, postnatal; and EPDS, Edinburgh postnatal depression scale (total scores).

**Table 11:** Predictors of maternal confidence and self-efficacy (MABS).

MABS	Variables	Unstandardised coefficients		Standardised coefficients	*t*	*p*
		*B*	*SEB*	*β*		
Global confidence	Constant	17.481	0.313		55.887	0.000
	Postnatal positive	0.215	0.063	0.114	3.445	0.001
	PN physical wellbeing	0.200	0.063	0.106	3.179	0.002
	Emotional stability	0.172	0.050	0.133	3.427	0.001
	EPDS total	−0.075	0.015	−0.206	−5.116	0.000
Lack of confidence in caretaking	Constant	30.836	0.770		40.052	0.000
	Number of children	−1.987	0.361	−0.166	−5.503	0.000
	EPDS total	0.650	0.053	0.369	12.253	0.000
Lack of confidence in breastfeeding	Constant	30.293	0.775		39.103	0.000
	Number of children	−1.824	0.359	−0.153	−5.081	0.000
	Meconium in waters	2.579	0.726	0.107	3.554	0.000
	EPDS total	0.624	0.053	0.357	11.828	0.000

B, unstandardised coefficient; SEB, standard error of unstandardised coefficient; and *β*, standardised beta.

The predominant predictors of maternal confidence were whether she had given birth before, known as parity (Number of Children), her subjective perinatal experience (e.g. Postnatal Positive) and psychological factors, such as EPDS and Emotional Stability (Table 11).

**Figure 1:**
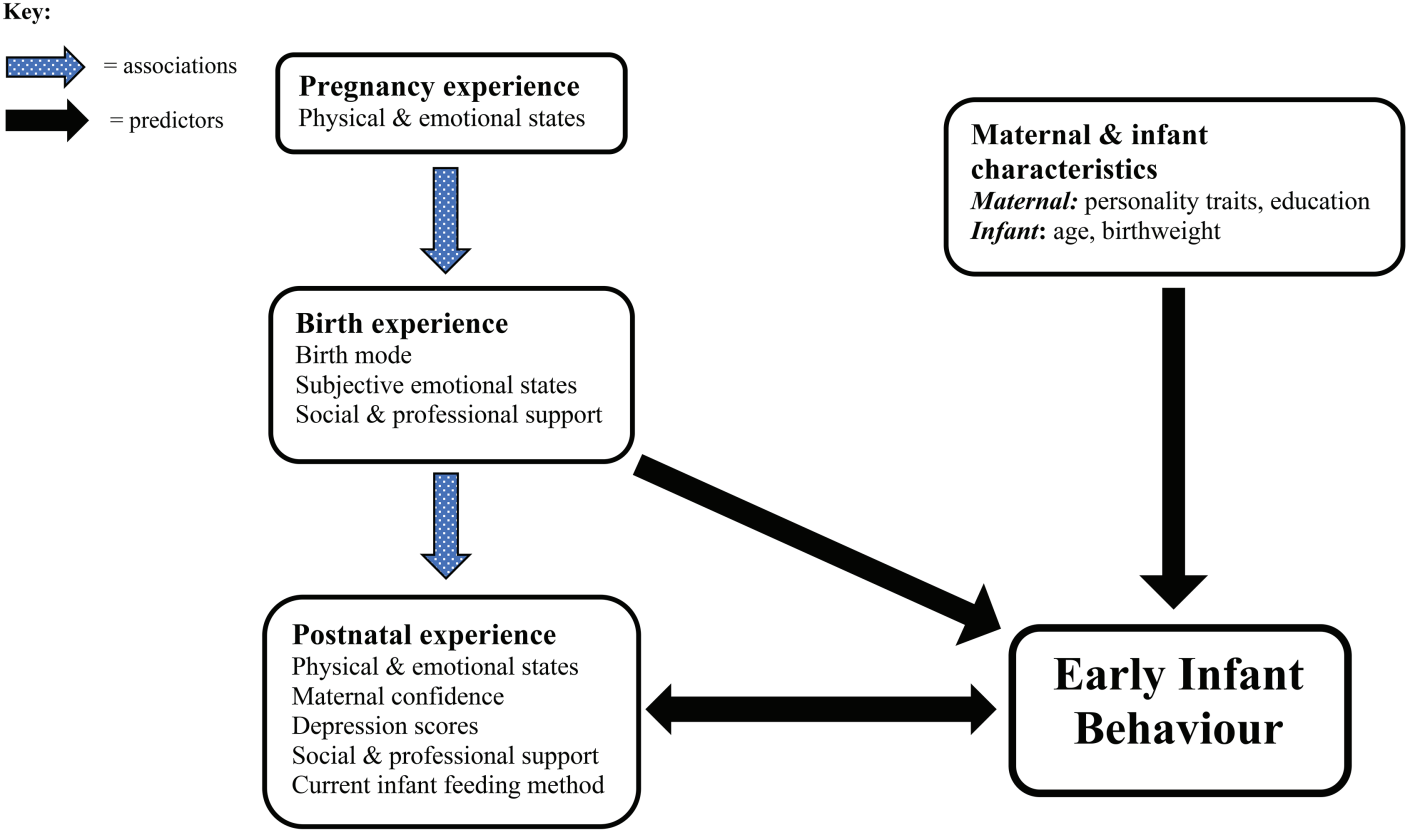
Representation of findings—perinatal factors contributing to early infant behaviour.

In summary, Figure 1 highlights the main factors identified as having the strongest associations and predictive values for infant behavioural outcomes. It emphasises the equal importance of physical and psychological factors at all stages of the perinatal journey and the ability of appropriate levels of social and professional support to shield and protect mother and infant from the immediate and potentially long-lasting impacts of a negative birth experience.

## Discussion

The aim of this study was to examine how maternal physical and psychological experiences of pregnancy, childbirth and the early postnatal period may be associated with the mother’s perceptions of her infant’s behaviour, taking into account broader maternal demographic factors, postnatal mood and personality. The study had several notable findings, and numerous aspects of childbirth and the perinatal period were associated with perceived early infant behaviour. Although certain childbirth interventions, complications, birth mode and types of pain relief were associated with less settled infant behaviour (0–6 months), regression analyses highlighted that subjective and psychological factors, alongside maternal personality traits, largely predicted perceived infant behavioural style and maternal confidence outcomes.

In addition, the mother’s sense of wellbeing during and post-birth was reflected in higher levels of reported confidence and self-efficacy alongside her perceptions of easier infant behaviour. The predictability of birth events could play a key role in situations where the mother’s sense of agency and control is increased or diminished, as research shows that agency in relation to free choice is particularly important for unpredicted negative events (Tanaka and Kawabata, 2019). More specifically, a Dutch study found that women were less happy with the care they received if they had experienced an instrumental vaginal (assisted) birth, an emergency C-section, less control during the active (dilation) stage or a more directed second (pushing) stage (Baas et al., 2017). This may help to explain the differences in maternal and infant responses between planned or unplanned C-sections in the present study. An elective C-section should involve at least an element of predictability, choice and a chance to prepare.

These findings support the concept that subjective maternal perceptions of childbirth may contribute to early infant temperament development or to maternal perceptions of her baby’s behavioural style. This could have important implications for maternity and midwifery practice, particularly for more vulnerable mother–infant pairs, or those who experience a challenging birth. The main findings are further discussed under the following headings.

### Physical Perinatal Factors

Although many of the more objective factors, such as interventions, birthplace, birth mode and gentle birth of the infant’s head, were associated with infant and maternal outcomes, breastfeeding was the only physiological perinatal factor that retained significance in the regression models for perceived infant behaviour. Specifically, ‘currently breastfeeding’ was associated with less Unsettled-Irregular behaviour over the first 6 months. Furthermore, infants were reported as less Alert during Feeds if they were currently breastfeeding, indicating that breastfeeding could be less stimulating and more relaxing for the infant. Breastfeeding is an instinctive newborn behaviour, highly sensitive to external stimuli from emotions, such as anxiety and fear (Moore et al., 2016). Its initiation and continuation could therefore be affected by the mother’s physical and emotional response to her birth and her new baby, including whether mother and newborn infant were able to have immediate skin-to-skin contact. Moreover, a mother feeling able to breastfeed her baby could signify that she had a more physiological birth with less pain relief (Widström et al., 2011; Brown and Jordan, 2013). Indeed, a systematic review by Uvnäs-Moberg et al. (2019) highlights the associations of spontaneous physiological birth experience with increased hormonal and physiological wellbeing of mother and infant post-birth, which is likely to make initiating and continuing breastfeeding easier.

This finding may also be connected to the close skin-to-skin contact that naturally occurs during breastfeeding, regulating hormonal systems after a challenging birth, enhancing oxytocin and lowering cortisol levels, reducing the negative impacts of pain and stress on neonatal and maternal HPA axes and benefitting mother–infant neurobiological wellbeing and synchrony post-birth (Carter, 2014; Feldman, 2015; Mooney-Leber and Brummelte, 2017).

### The Psychological Birth Experience

Consistent with a meta-analysis highting how maternal perceptions of a traumatic birth may contribute more to symptoms of CB-PTSD than objective physical factors, such as birth mode (Ayers et al., 2016), we found that self-reported infant behaviour was largely predicted by psychological and subjective maternal factors. However, this finding could also indicate that strong psychological variables, such as postnatal depression, may override other contributory factors (Leigh and Milgrom, 2008). Indeed, EPDS scores were the strongest predictor of several infant and maternal outcome variables including unsettled, irritable and irregular infant behaviour alongside lower general maternal confidence as well as confidence in caretaking and breastfeeding. These findings could mean that depressed mothers perceive their infant as more difficult (McGrath et al., 2008). Furthermore, they may indicate that the mother’s personal feelings of birth trauma, represented here by negative birth and postnatal emotional states, such as Anxious-Afraid, Neglected and Postnatal Distress, contribute equally to PPD and CB-PTSD, with an adverse impact on mother–infant bonding (Stuijfzand et al., 2020), in turn affecting infant temperament. Alternatively, a challenging birth experience could lead directly to increased unsettled infant behaviour *via* physiological pathways, as discussed. This may result in lower maternal confidence combined with more negative thoughts and feelings. Thus, the relationship between mother and infant states of wellbeing or otherwise is likely to be bi-directional (see Figure 1 above).

Also consistent with Ayers et al. (2016), correlational analyses here showed that certain subjective factors, such as Anxious-Afraid, Postnatal Distress and EPDS scores, were directly related to more interventionist birth modes. Therefore, these findings provide further support for a pathway between obstetric interventions, lack of control, negative birth emotions and postpartum mood disturbances (Blom et al., 2010; Ayers et al., 2016; Field, 2017). In turn, negative postnatal maternal mood may adversely impact on mother–infant interactions and subsequent infant behaviour and development (Murray et al., 2018). Our results show that maternal interpretations of the birth could be equally important to more objective measures, such as birth mode and obstetric complications and interventions.

### How Maternal Wellbeing Might Be Associated With Infant Behaviour

There are several potential pathways for how the mother’s birth experience, maternal wellbeing and infant behaviour could be interacting with one another.

#### Maternal Psychological Experience of Birth

Mothers who experienced a difficult birth were more likely to report feeling Anxious-Afraid or Neglected (‘ignored’ or ‘abandoned’) during childbirth and distressed postnatally, and these negative emotional states were associated with maternal reports of Unsettled-Irregular infants. As speculated by prior research (Douglas and Hill, 2013), this may be connected to the interacting hormonal systems of mother and infant. Higher cortisol levels can dysregulate the HPA axis, potentially causing long-term changes to the infant microbiome and epigenome (Dahlen et al., 2013, 2014; Almgren et al., 2014), with consequences for future behaviour and development (Gitau et al., 2001; Wolke et al., 2009; Schmid et al., 2010; Prokasky et al., 2017).

In contrast, having a more ‘supported’ experience and feeling positive post-birth predicted higher scores for Alert-Responsive infant behaviour and overall perceived infant Easiness. These mothers rated their midwife as helpful and informative and felt ‘emotionally supported’ throughout, reflecting evidence that mothers experience less anxiety post-childbirth if they feel well cared for during the birth (Field, 2017). This pathway could occur physiologically *via* an easier birth and maternal recovery, with the mother’s wellbeing during and post-birth encouraging neonatal wellbeing *via* their inter-connected hormonal systems (Buckley, 2015; Buckley and Uvnäs-Moberg, 2019). Alternatively, a mother who experiences positive birth emotions and therefore increased levels of oxytocin and beta-endorphins, may simply *perceive* her newborn infant more positively. Subjective maternal response to childbirth may therefore be a factor in the mother’s own postnatal wellbeing (Ayers et al., 2016) and her subsequent perceptions of and interactions with her baby (Murray et al., 2014, 2018) as well as affecting the infant’s behavioural response (Taylor et al., 2000).

#### Postnatal Mood

Higher maternal postnatal depression (EPDS) scores predicted perceptions of unsettled, irregular and irritable infant behavioural style, supporting previous research identifying a link between postnatal depression and perceptions of more ‘difficult’ infant behaviour (Gonidakis et al., 2008; Britton, 2011). This relationship is likely to be bi-directional: a crying, irritable infant may affect maternal mood, exacerbated by sleep deprivation (Eastwood et al., 2012), and an infant may become unsettled in response to negative maternal mood (Martini et al., 2017).

EPDS postnatal depression scores were associated with maternal Postnatal Distress, which in turn was associated with both the physical and emotional birth experience, aligning with evidence that negative birth experiences and postnatal psychological states contribute to postpartum depression (Bell and Andersson, 2016). Moreover, a difficult or interventionist birth might lead directly to increased unsettled infant behaviour; and excessive infant crying predicts later EPDS scores, particularly if the mother feels unable to console her baby (Radesky et al., 2013).

Oxytocin promotes bonding and attachment (Feldman, 2017; Uvnäs-Moberg et al., 2019). Conversely, depressed mothers with lower oxytocin levels are more likely to ignore their infant’s cues (Mah et al., 2017). Consequently, postnatal depression and maternal withdrawal are associated with interactional difficulties, affecting mother–infant bonding and infant outcomes even after maternal mood improves (Murray et al., 2014, 2018; Oyetunji and Chandra, 2020).

#### Maternal Personality

As expected, heritable maternal character traits predicted self-reported infant behavioural style and maternal confidence ratings. The mother’s personality influences outcomes on three levels: how she feels, the way she responds to her newborn baby and the postnatal environment she creates (Carey and McDevitt, 2016). Therefore, the impact of maternal personality on infant behaviour occurs through a combination of genetic and environmental influences. Although the personality trait ‘Emotional Stability’ was not retained as a predictor variable for perceived infant behaviour, it predicted Global Confidence and was inversely related to EPDS scores which came through as a strong predictor of Unsettled-Irregular infant behaviour over the first 6 months. Thus, maternal mood, personality and infant temperament were reciprocally associated.

#### Birth Companions

A less anticipated finding was that the presence of an extra birth companion alongside the birth partner positively predicted maternal perceptions of infant Easiness. Potentially, having two continuously supportive figures in the birthing room contributes more effectively to a positive birth experience with positive outcomes, including enhanced maternal perceptions of her baby. Furthermore, we know that continuous emotional support from a female companion, such as a doula, may lessen maternal stress levels, boost oxytocin (Buckley, 2015) and encourage a shorter labour and a normal birth, with lower use of analgesia and higher infant Apgar scores (Bohren et al., 2017). Decades of research illustrate the positive psychological impacts of doula support (Sosa et al., 1980; Kennell et al., 1991; Bohren et al., 2017). For instance, mothers accompanied in labour by a doula are known to have increased confidence in caretaking, lower levels of depression and to think more positively of their infants (Klaus and Kennell, 1997).

#### Sociodemographic Status and Maternal Expectations of Motherhood

Finally, sociodemographic variables were associated with certain types of infant behaviour. Mothers who did not attend higher education perceived their infants as more alert, responsive and easier overall. These findings might be explained through examining previous research which shows that mothers with higher education in established careers can find the transition to motherhood more challenging and have lower life satisfaction after having a baby (Harwood et al., 2007). This general negative mood could in turn affect the mother’s perceptions of her baby’s behaviour (McGrath et al., 2008). Furthermore, parents of higher social classes may have higher expectations of their children’s future (Lareau, 2011; Irwin and Elley, 2013). Unrealistic expectations of their baby could result in disappointment and contribute to depressive symptoms (Martin et al., 2013), in turn negatively impacting on maternal confidence and ability to bond with her infant.

### Strengths and Limitations

This study provided an in-depth exploration of a concept previously only alluded to in the research literature on childbirth and infant behaviour: that potentially long-term physiological impacts of childbirth on the infant’s behavioural style and stress response system (Douglas and Hill, 2013) could possibly be mediated by the mother’s subjective response to the birth (Taylor et al., 2000). Indeed, our findings showed that the mother’s subjective response to the birth affected her perceptions of her baby’s behaviour more than the objective physical experience. However, care needs to be taken between statistical and clinical significance, particularly where effect sizes are small. Quantitative psychological data often has this issue, in part due to the common inter-correlation of psychological variables (Field, 2009), the difficulty in separating out such variables and thus the small individual contribution that each one finally makes to the overall picture. Nevertheless, as a whole and often supplemented by qualitative data (e.g. Power et al., 2019), the summative effect of multiple small statistical differences for different though inter-related physical and psychological variables (such as birth mode and postnatal psychological wellbeing/distress) might make an actual difference to real life experiences. Therefore, they become meaningful to maternity and perinatal care in the context of the other research evidence in this area.

Nevertheless, this research is not without limitations. First, while the online nature of the study allowed for a nationwide data collection strategy, this increasingly popular research method may contain drawbacks, especially in equity of recruitment. For instance, non-native English speakers or those without a good level of written English may have felt intimidated by the length of the survey as well as the language contained within the survey. To encourage maximum inclusivity, questions were made as straightforward and self-explanatory as possible with the majority of responses (except for, e.g. birth weight) recorded *via* check boxes. However, ethnic minority use of the Internet is slightly below average (Gov.uk, 2019). This may have contributed to a mostly white Caucasian sample population, which was also skewed toward breastfeeding, older mothers who were living with a partner. Despite this, and although an online survey did not cater for women without Internet access due to factors such as socio-economic deprivation, reportedly most women of childbearing age in the United Kingdom have access to the Internet (Gov.uk, 2019). There was a wide variation in participants’ socio-economic status and women from a diverse range of socio-economic groups took part in the survey.

There are drawbacks to employing a self-report retrospective survey linked to the issue of subsequent validity of findings in regard to accurate recall of autobiographical memories (Belli, 1998). However, having had a period of reflection since an emotive or anxious birth situation might in fact aid more accurate recall. Retrospective questionnaires have become a popular, cost-effective and acceptable method of collecting pregnancy and childbirth data (Intong et al., 2017). Moreover, questionnaires about childbirth have been found to have excellent validity during the first few months post-birth (Bat-Erdene et al., 2013).

Retrospective reports of infant behaviour could be affected by biased memories of events and current behaviours during periods involving growth spurts, teething or weaning, all of which may alter the infant’s normal behavioural patterns. They could also be influenced by maternal mood, although this effect is considered small (McGrath et al., 2008). Maternal ratings of infant behavioural scores are significantly associated with trained observer ratings (Rothbart et al., 2001; Henderson and Wachs, 2007; Zentner and Bates, 2008). Thus, an infant behaviour questionnaire was chosen that focused on specific everyday infant behaviours over the past 7 days (MABS), and the survey was completed within 6 months of the birth.

As this was a cross-sectional study, a future prospective longitudinal study could measure the same sample of women and infants at various time points throughout their perinatal experience. Importantly, the sample population was self-selecting. This undoubtedly affected the type of participant who completed the online questionnaire, rendering the recruitment strategy less inclusive. Potentially, however, a more homogeneous sample could help clarify findings in terms of specific impacts of childbirth experience on perceived infant behaviour.

**Figure 2:**
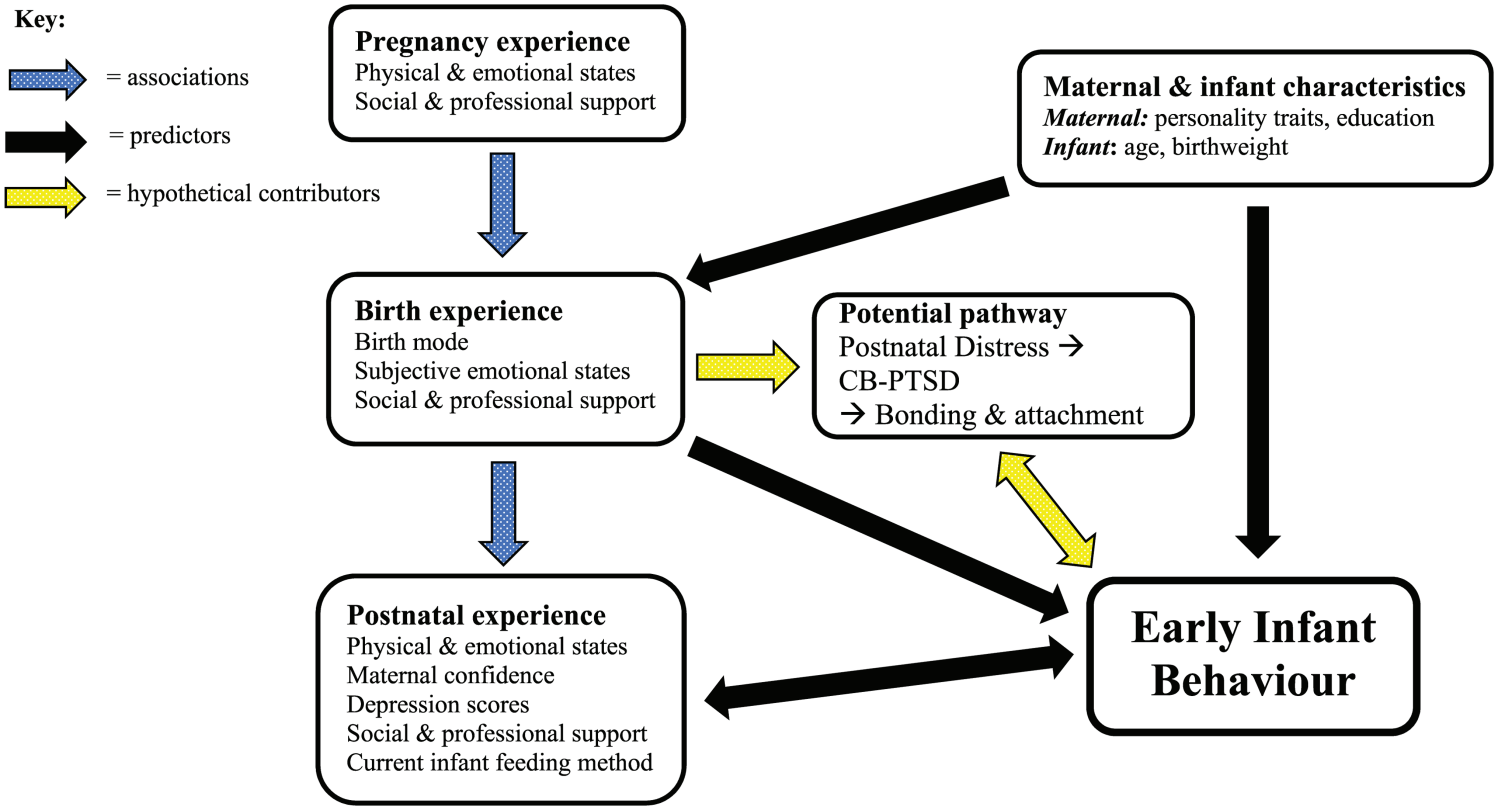
A theoretical model of perinatal factors contributing to early infant behaviour.

Given that these findings provide support for those of a previous qualitative study, with health professional data collected over a similar period (Power et al., 2019), a future study should include a measure of perceived birth trauma and CB-PTSD symptoms as well as a scale for mother–infant interactions and bonding–attachment behaviours. This may provide a more complete picture, such as that suggested by Figure 2. The ‘hypothetical contributors’ shown in Figure 2 represent items for future testing based on the findings in this study, the aforementioned health professional data and the wider research literature.

Difficulties in bonding and attachment processes could be a key point in this connection between the mother’s response to childbirth (e.g. Postnatal Positive/Distress) and interpretations of her baby’s behaviour. Stuijfzand et al. (2020) study showed that maternal distress at 1 month postpartum—which was associated with a traumatic birth experience—adversely impacts mother–infant bonding at 3 months postpartum. These authors also highlighted antenatal support in the pathway to a positive or negative birth experience and CB-PTSD. Similarly, Davies et al. (2008) found associations between CB-PTSD symptoms post-childbirth and more negative maternal perceptions of her infant alongside lower attachment; and a large Internet survey of mothers giving birth during the COVID-19 pandemic found that acute stress during childbirth had adverse impacts on mother–infant bonding and breastfeeding (Mayopoulos et al., 2021).

Despite their possible contribution to infant behavioural outcomes, no direct measures were used here to assess symptoms of CB-PTSD or bonding and attachment behaviours between the mother and her baby. Nevertheless, the suggested pathway in this study between maternal birth experience and perceived infant behavioural style—*via* the influences of postpartum maternal mood on mother–infant bonding and attachment—warrants further investigation. To include measures of CB-PTSD symptomology alongside early mother–infant interactions and bonding would therefore add to a more complete theoretical model for future testing (see Figure 2). In line with Stuijfzand et al. (2020) findings, professional and social support during pregnancy have been added to this model. Thus, Figure 2 aims to provide a broader picture of the potential mechanisms behind the associations found in this study between maternal childbirth experience and infant behavioural style. The ‘Potential pathway’ box illustrates how a negative birth experience may be part of a pathway involving (maternal) Postnatal Distress, symptoms of CB-PTSD, bonding and attachment issues and maternal perceptions of more difficult, unsettled infant behaviour.

Our results add to a large body of research illustrating the complexity of childbirth and its potential outcomes for mother and infant. They highlight how infant and maternal outcomes of childbirth appear to be mutually influenced by one another’s response to birth and by multiple physiological and psychological perinatal variables, such as feeling anxious and afraid or neglected during childbirth. Social and professional support the mother receives during the perinatal period may positively enhance her response to childbirth, in turn benefitting her infant’s behavioural style. This finding is particularly pertinent in light of United Kingdom maternity policies involving restrictions on home births, water births and partner accompaniment in early labour during the COVID-19 pandemic. In some areas, this led to an increase in women opting for ‘free-births’ without any professional care—often to keep their partner with them throughout labour and birth and, for others, to avoid catching COVID (Feeley et al., 2021). Restrictions on partner accompaniment during early labour also led to more women accidentally giving birth alone in hospital settings if there happened to be staff shortages and labour was unexpectedly quick (Feeley et al., 2021).

Our findings therefore provide further support for the current United Kingdom maternity and midwifery services’ objective to increase staffing numbers, reduce risk and promote a model of safe, consistent, continuous and emotionally supportive care for all expectant mothers (NHS England National Maternity Review, 2016; Scottish Government, 2017; The Regulation and Quality Improvement Authority, Northern Ireland, 2017; Healthcare Inspectorate Wales, 2020). Overall, these United Kingdom-wide reviews emphasise that health care should be both individualised and family centred with a focus on equity of care and informed choice.

The most recent of these maternity reviews—namely the Welsh review (Healthcare Inspectorate Wales, 2020)—although notably its investigations were carried out *pre*-March 2020, observed that maternity services have been very stretched during the COVID-19 pandemic. These problems arising in maternity care across the United Kingdom are intensifying previous issues of staff shortages and a lack of emotionally supportive care and are leading to increased reports of psychological distress in new mothers (Alcindor, 2021). Considering the impacts of the sudden reduction in face-to-face support for mothers and their babies during the early pandemic (Silverio et al., 2021), which is still occurring in some areas of maternity and perinatal care, it is vital that these services are made a priority in the government’s plans to ‘build back better’. Following the MBRACE report (MBRACE-UK, 2020), an urgent emphasis must especially be placed on equity of care. Thus, both physical and psychological wellbeing during and after childbirth need to become the objective for all mothers and their infants.

Maternity research collaboratives, including The Lancet Midwifery Series (ten Hoope-Bender et al., 2014) and the European Cooperation in Science and Technology (COST, 2020), have highlighted the importance of promoting and valuing high-quality and compassionate midwifery and newborn care for this all-important mother–infant health and wellbeing. This notion of ‘quality’ midwifery care was set out by ten Hoope-Bender et al. (2014) to include providing preventative, respectful and supportive care to women and their infants, swift medical treatment where required and using medical interventions only when clinically indicated. Consistent with our findings, research evidence around the importance of facilitating optimal neurohormonal states during physiological labour and birth emphasises the inter-connectedness of psychosocial and physiological factors for positive birth outcomes (Downe et al., 2020; Olza et al., 2020). Aligning with prior evidence around the significance of mother–infant neurobiological wellbeing and synchrony post-birth (Carter, 2014; Feldman, 2015; Mooney-Leber and Brummelte, 2017), our findings show how a positive birth experience enhances postnatal maternal mood and the mother’s perceptions and experiences of her baby’s early temperament, encouraging a happier and more fulfilling long-term relationship for both.

## Conclusion

As recommended by the WHO (2018, p. 1) in their intrapartum guidelines supporting women’s right to a ‘*positive birth experience*’, this should include ‘*giving birth to a healthy baby in a clinically and psychologically safe environment with continuity of care and emotional support*’. Promoting maternal emotional wellbeing alongside physical safety during and after childbirth is of paramount importance. High-quality one-to-one midwifery care during childbirth may benefit the mother’s physiological and psychological states (Olza et al., 2020) and consequently enhance her perceptions and experiences of her baby’s behaviour. Conceivably, protecting the mother’s neurohormonal state during childbirth and postnatally could also help to protect her against postpartum mood disorders and in this way promote more sensitive parenting and increase mother–infant bonding behaviours, with a positive impact on infant socio-emotional and cognitive development (Murray et al., 2014, 2018; Field, 2017; Tichelman et al., 2019). This could benefit not only the mothers and infants themselves but also their families and the wider society in which they live.

## Data Availability Statement

The raw data supporting the conclusions of this article will be made available by the authors, without undue reservation.

## Ethics Statement

The studies involving human participants were reviewed and approved by Swansea University Department of Psychology Research Ethics Committee. The patients/participants provided their written informed consent to participate in this study.

## Author Contributions

CP: design, conceptualisation, methodology, data collection, formal analysis, writing—original draft, writing—review and editing, and project administration. CW and AB: design, conceptualisation, methodology, data analysis support, writing—review and editing, and supervision. All authors gave approval for submission, contributed significantly to the article, and are responsible for its contents.

## Conflict of Interest

The authors declare that the research was conducted in the absence of any commercial or financial relationships that could be construed as a potential conflict of interest.

## Publisher’s Note

All claims expressed in this article are solely those of the authors and do not necessarily represent those of their affiliated organizations, or those of the publisher, the editors and the reviewers. Any product that may be evaluated in this article, or claim that may be made by its manufacturer, is not guaranteed or endorsed by the publisher.
